# Correlates of Unsupervised Bathing of Infants: A Cross-Sectional Study

**DOI:** 10.3390/ijerph10030856

**Published:** 2013-03-04

**Authors:** Mirjam E. J. van Beelen, Eduard F. van Beeck, Paul den Hertog, Tinneke M. J. Beirens, Hein Raat

**Affiliations:** 1 Department of Public Health, Erasmus MC-University Medical Center, P.O. Box 2040, Rotterdam 3000 CA, The Netherlands; E-Mails: m.vanbeelen@erasmusmc.nl (M.E.J.B.); e.vanbeeck@erasmusmc.nl (E.F.B.); t.beirens@erasmusmc.nl (T.M.J.B.); 2 Consumer Safety Institute, P.O. Box 75169, Amsterdam 1070 AD, The Netherlands; E-Mail: p.denhertog@veiligheid.nl; 3 Dutch Association for Youth Health Care Physicians, Churchillaan 11, Utrecht 3527 GV, The Netherlands

**Keywords:** children, supervision, bathing, drowning, safety, This study was registered in the Netherlands National Trial Register (NTR), Trial Registration Number NTR1836.

## Abstract

Drowning represents the third leading cause of fatal unintentional injury in infants (0–1 years). The aim of this study is to investigate correlates of unsupervised bathing. This cross-sectional study included 1,410 parents with an infant. Parents completed a questionnaire regarding supervision during bathing, socio-demographic factors, and Protection Motivation Theory-constructs. To determine correlates of parents who leave their infant unsupervised, logistic regression analyses were performed. Of the parents, 6.2% left their child unsupervised in the bathtub. Parents with older children (OR 1.24; 95%CI 1.00–1.54) were more likely to leave their child unsupervised in the bathtub. First-time parents (OR 0.59; 95%CI 0.36–0.97) and non-Western migrant fathers (OR 0.18; 95%CI 0.05–0.63) were less likely to leave their child unsupervised in the bathtub. Furthermore, parents who perceived higher self-efficacy (OR 0.57; 95%CI 0.47–0.69), higher response efficacy (OR 0.34; 95%CI 0.24–0.48), and higher severity (OR 0.74; 95%CI 0.58–0.93) were less likely to leave their child unsupervised. Since young children are at great risk of drowning if supervision is absent, effective strategies for drowning prevention should be developed and evaluated. In the meantime, health care professionals should inform parents with regard to the importance of supervision during bathing.

## 1. Introduction

Drowning is a major health problem among children around the globe [1]. Drowning refers to an event in which a child’s airway is immersed in a liquid medium, leading to difficulty in breathing [2]. This event may result in death or (permanent) disability. In the United States, drowning represents the third leading cause of fatal unintentional injury in infants less than one year of age, and over half of these fatal drownings occur in bathtubs [3,4]. Up to the age of twelve months infants may be able to sit up unsupported, although some are still unable to right themselves if they fall over in the bathtub [5,6]. At young age, infants fully rely on their parents to prevent potential harm.

Adequate child supervision is likely to be the most effective defense against many childhood unintentional injuries [7]. However, still many injuries to young children, for example drowning, poisoning, or falls, occur in their homes when caregivers are responsible for a safe environment and adequate supervision [8]. It has been shown that parents’ opinions about the necessity of adult supervision are associated with parent behavior and family and household characteristics [7]. In acute poisoning, an increased number of children in the home is associated with less parental supervision related to the presence of older children [9]. So far, little is known about the correlates of unsupervised bathing, although in over three quarters of bathtub drownings, lack of adequate adult supervision is involved [10,11]. Two previous studies found that some parents believe they can leave their infant unsupervised in the bathtub for a short moment [3,12]. One single study showed that many parents fail to realize the severity of drowning [13].

To develop strategies for infant drowning prevention, more insight is needed into the correlates of unsafe behaviour. In addition to the role of socio-demographic characteristics, insight with regard to psychosocial constructs of unsupervised bathing is needed, using a theoretical model. Protection Motivation Theory (PMT) is a framework particularly suited for interventions of protective, precautionary behaviours [14]. PMT is suggested to be applied to assess the influence of psychosocial factors on parental safety behaviour. According to PMT, the probability of health protective behaviour or an “adaptive response”—in this case supervision during bathing—is increased by four factors: (1) the threat is perceived as severe (severity); (2) the threat is perceived as high of personal relevance (vulnerability); (3) the adaptive response is perceived as affective for warding off the threat (response efficacy); and (4) the personal abilities and self-confidence to engage in the adaptive response is perceived as high (self-efficacy). The aim of this study is thus to investigate correlates of unsupervised bathing.

## 2. Experimental Section

### 2.1. Participants and Setting

The present cross-sectional study used data obtained at enrolment in the “BeSAFE” study, a randomized controlled trial which aims to assess the effects of internet-based, tailored safety information combined with personal counselling on parents’ child safety behaviours, as described in detail elsewhere [15]. The “BeSAFE” study addressed several topics with regard to the safety in and around the home, such as the prevention of falling, poisoning, drowning and burning. The Medical Ethics Committee of the Erasmus Medical Center gave a “declaration of no objection” for this study (MEC-2008-370).

Parents of infants (4–12 months), attending a regular well-child visit were invited by their child health care professional to participate in a home safety survey on multiple home safety topics. An opportunity sample of five child health care organizations, located in both urban and rural areas of the Netherlands, invited a total of 3,147 parents between 2009 and 2010. A total of 1,440 parents (45.8%) provided informed consent for the baseline questionnaire and completed the baseline questionnaire (in Dutch). Parents who did not wish to participate in the follow-up of the “BeSAFE” study were invited to anonymously complete the baseline questionnaire. Sixty parents completed the baseline questionnaire anonymously and 1,380 parents provided informed consent for participation in the complete “BeSAFE” study. A total of 30 records were removed, because parents did not bathe their child. A study population of 1,410 parents and children was complete for data analysis.

### 2.2. Design

Parents received written information about the study, were asked to provide informed consent and complete the baseline questionnaire on home safety. The baseline questionnaire included questions on socio-demographic factors, safety behaviour and PMT constructs.

### 2.3. Parental Supervision

Parental supervision of their infant during bathing was assessed by the item asking parents “How often do you leave your child alone when he/she is in the bathtub, even just for a short time?”; answering very often/often/sometimes/rarely/never.

### 2.4. Potential Correlates

We used socio-demographic variables and PMT constructs to investigate potential correlates of supervision during bathing. The socio-demographic variables, age, gender, number of parents and siblings, and parental employment, education and ethnicity, included in this study were chosen based on earlier studies reporting the influence of these variables on safety behaviours [16,17,18].

Ethnicity of the parents (Dutch or Western migrant; non-Western migrant) was determined on the basis of grandmothers’ and grandfathers’ country of birth according to the definitions of Statistics Netherlands [19]. The parent was of non-Western ethnic origin if at least one of their parents was born in a non-Western country. If both their parents were born in a non-Western country, ethnicity was determined according to the mother’s country of birth.

Crawling was defined as the child being able to: “crawl on hands and knees and/or crawl on their tummy and/or shuffle on their bottom” (yes/no).

Additionally, psychosocial constructs were measured within the domain of PMT. All items related to PMT were measured on bipolar five-point scales. Self-efficacy was measured by the item asking parents how difficult or easy they perceive taking the safety measures to be (from −2 = very difficult to +2 = very easy). Response efficacy was measured by the item assessing how helpful parents perceived the specific behaviour to be for preventing an injury (from −2 = not very helpful to +2 = very helpful). Vulnerability was measured by the item asking parents their perception of their child’s risk of an unintentional injury on each specific subject (from −2 = low risk to +2 = high risk). Severity was measured by the item assessing how seriously parents perceived the consequences of an injury occurring in the bathtub (from −2 = not serious at all to +2 = very serious).

### 2.5. Statistical Analyses

Statistical analyses were performed using SPSS 17.0 (SPSS Inc., Chicago, IL, USA). Level of supervision was dichotomized into leaving the child unsupervised (very often/often/sometimes/rarely) and never leaving the child unsupervised. Frequency tables were used to explore the socio-demographic characteristics of the total study population, and those categorized as left unsupervised and never left unsupervised. Mean and frequency differences between children who were left unsupervised and children who were never left unsupervised were evaluated through independent sample *t*-tests and Chi-square statistics, respectively.

Correlations were calculated to assess multicollinearity. All correlations were under 0.60, indicating that multicollinearity would not be an issue for multiple logistic regression analyses.

To determine significant correlates of parents who leave their infant unsupervised, three steps of logistic regression analyses were performed with supervision during bathing (left unsupervised/never left unsupervised) as the dependent variable and potential correlates (socio-demographic and PMT constructs) as independent variables. In Model 1 the potential correlates were entered univariate. Secondly, a multiple model was constructed using a manual-enter selection method in which all independent socio-demographic variables were included. Subsequently all variables with the highest *p* value were deleted from the model, until all variables had a *p* value of 0.05 or less. In Model 3 a multiple model with socio-demographic variables and PMT constructs were included, using the same stepwise backward analyses as performed in Model 2.

## 3. Results and Discussion

### 3.1. Participants

The mean age of the children was 7.2 months (SD 1.1; range 4–12 months); 48.3% were girls; 34.4% could crawl. Of the participating families 93.3% of the mothers completed the questionnaire and 48.4% had one child (Table 1).

**Table 1:** Child and family characteristics and PMT constructs of supervision of infants in bathtubs (n = 1,410).

	Total group	Children left unsupervised	Children never left unsupervised	*p* value
Infant is bathed in bathtub	n = 1,410	n = 87	n = 1,319	
**Child characteristics**	**n (%)**	**n (%)**	**n (%)**	
*Mean age in months (SD)*	7.2 (1.1)	7.5 (1.2)	7.2 (1.1)	0.01 **
*Range in months*	4–12	4–12	4–12	
*Gender*				
Girl	681 (48.3)	47 (54.0)	633 (48.0)	0.28 *
*Child can crawl*				
Yes	484 (34.4)	34 (39.1)	450 (34.2)	0.35 *
**Family characteristics**	**n (%)**	**n (%)**	**n (%)**	
***Mother is respondent***	1,315 (93.3)	83 (95.4)	1,229 (93.2)	0.71 *
***Family situation***				
One-parent family	39 (2.8)	3 (3.5)	36 (2.7)	0.67 *
***Number of children in family***				
One child	682 (48.4)	34 (39.1)	645 (48.9)	0.08 *
Two or more children	728 (51.6)	53 (60.9)	674 (51.1)	
***Mother***				
*Employment*				
Paid job fulltime	90 (6.6)	7 (8.5)	83 (6.5)	0.63 *
Paid job part time	1,024 (75.1)	63 (76.8)	959 (75.1)	
No paid job	249 (18.3)	12 (14.6)	235 (18.4)	
*Educational level*				
Low	234 (16.6)	11 (12.6)	222 (16.9)	0.51 *
Intermediate	623 (44.2)	38 (43.7)	583 (44.3)	
High	551 (39.1)	38 (43.7)	512 (38.9)	
*Ethnicity*				
Western	1,292 (91.6)	79 (90.8)	1,211 (91.8)	0.55 *
Non Western	118 (8.4)	8 (9.2)	108 (8.2)	
***Father***				
*Employment*				
Paid job fulltime	1,149 (85.7)	70 (87.5)	1,076 (85.6)	0.81 *
Paid job part time	138 (10.3)	6 (7.5)	131 (10.4)	
No paid job	54 (4.0)	4 (5.0)	50 (4.0)	
*Educational level*				
Low	319 (22.9)	23 (27.1)	295 (22.6)	0.62 *
Intermediate	569 (40.8)	32 (37.6)	536 (41.1)	
High	506 (36.3)	30 (35.3)	474 (36.3)	
*Ethnicity*				
Western	1,284 (91.8)	81 (95.3)	1,200 (91.6)	0.30 *
Non-Western	115 (8.2)	4 (4.7)	110 (8.4)	
**PMT constructs**	**Mean (SD)**	**Mean (SD)**	**Mean (SD)**	
Self-efficacy (−2, +2)	1.49 (1.0)	0.57 (1.0)	1.56 (0.9)	<0.0001 **
Response efficacy (−2, +2)	1.74 (0.5)	1.16 (0.7)	1.77 (0.5)	<0.0001 **
Vulnerability (−2, +2)	−1.42 (0.9)	−1.21 (0.8)	−1.43 (0.9)	0.02 **
Severity (−2, +2)	1.39 (0.9)	0.92 (1.0)	1.42 (0.9)	<0.0001 **

* Chi-square-test. ** Independent sample *t*-test. Missing values were 4 for infant is bathed; 1 for child’s gender; 3 for respondent’s gender, 4 for number of children; 47 for mother’s employment; 4 for mother’s educational level; 4 for mother’s ethnicity; 69 for father’s employment; 16 for father’s educational level; and 11 for father’s ethnicity.

Of the parents, 6.2% left their child unsupervised in the bathtub (4.8% rarely, 1.2% sometimes, and 0.2% (very) often).

**Figure 1:**
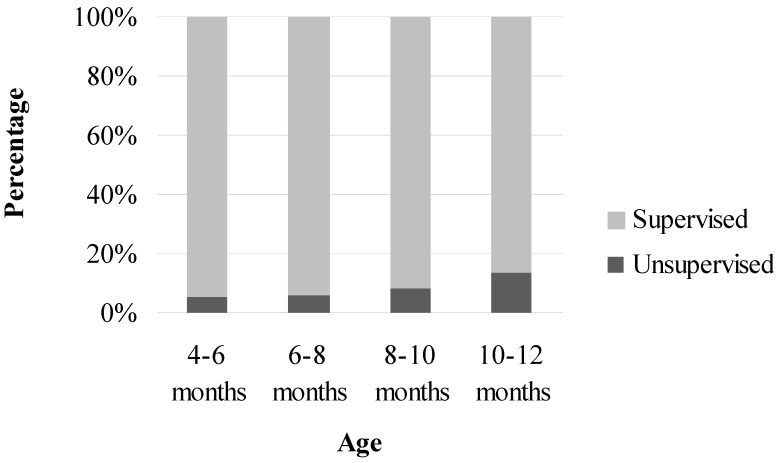
Percentage of children left unsupervised by age group.

The percentage of children who were left unsupervised in the bathtub rose with increasing age; 5.1% of children aged 4–6 months were left unsupervised; 5.7% of children aged 6–8 months were left unsupervised; 8.1% of children aged 8–1 0 months were left unsupervised and 13.3% of children aged 10–12 months were left unsupervised (Figure 1).

Parents with only one child left their infant less often alone in the bathtub compared to parents with two or more children (*p* = 0.08). Compared to parents who never left their child unsupervised in the bathtub, parents who left their child unsupervised had lower self-efficacy; mean 0.57 (SD 1.0) *vs.* 1.56 (SD 0.9), reported higher vulnerability; mean −1.21 (SD 0.8) *vs.* −1.43 (SD 0.9), perceived lower severity; mean 0.92 (SD 1.0) *vs.* 1.42 (SD 0.9), and reported lower response-efficacy; mean 1.16 (SD 0.7) *vs.* 1.77 (SD 0.5) (all *p* < 0.05; Table 1).

### 3.2. Correlates of Leaving the Infant Unsupervised in the Bathtub

Table 2 presents results of the univariate and multiple logistic regression analyses.

In the first model, age, self-efficacy, response efficacy, vulnerability, and severity were significant correlates of supervision of their infant (*p* < 0.05).

In the second model only age was significantly associated with leaving the infant unsupervised in the bathtub (OR 1.29; 95%CI 1.06–1.57).

In the subsequent step, when PMT constructs were added to Model 2 (Model 3), a model with six correlates was significant (*p* < 0.05). With rising age of the child, the more likely parents were to leave their child unsupervised in the bathtub (OR 1.24; 95%CI 1.00–1.54). Parents with one child were less likely to leave their child unsupervised in the bathtub (OR 0.59; 95%CI 0.36–0.97). Fathers of non-Western ethnicity were less likely to leave their infant unsupervised than fathers of Western ethnicity (OR 0.18; 95%CI 0.05–0.63). Parents who leave their infant unsupervised in the bathtub reported significantly less self-efficacy (OR 0.57; 95%CI 0.47–0.69), response efficacy (OR 0.34; 95%CI 0.24–0.48) and perceived severity (OR 0.74; 95%CI 0.58–0.93).

**Table 2:** Odds ratios (OR) and 95% confidence intervals from univariate (Model 1) and multiple logistic regression analyses with leaving the infant unsupervised in the bathtub as dependent variable and demographic variables (Model 2) and Protection Motivation Theory (PMT) variables (Model 3) as independent factors (n = 1,410).

	Infant left unsupervised in the bathtub					
	Model 1 OR (95%CI)	*p* value	Model 2 OR (95%CI)	*p* value	Model 3 OR (95%CI)	*p* value
**Demographic variables**						
***Infants***						
Age in months	**1.29 (1.06–1.57)**	**0.01**	**1.29 (1.06–1.57)**	**0.01**	**1.24 (1.00–1.54)**	**0.05**
Girl	1.27 (0.82–1.97)	0.28	-	-	-	-
Infant can crawl	0.81 (0.52–1.26)	0.35	-	-	-	-
***Family***						
One-parent family	1.30 (0.39–4.30)	0.67	-	-	-	-
First-time parent	0.67 (0.43–1.05)	0.08	-	-	**0.59 (0.36–0.97)**	**0.04**
***Mother***						
*Employment*						
Paid job fulltime	1.00	-	-	-	-	-
Paid job part time	0.78 (0.35–1.76)	0.55	-	-	-	-
No paid job	0.61 (0.23–1.59)	0.31	-	-	-	-
*Educational level*						
Low	1.00	-	-	-	-	-
Intermediate	1.32 (0.66–2.62)	0.44	-	-	-	-
High	1.50 (0.75–2.98)	0.25	-	-	-	-
*Non-Western migrant*	1.14 (0.54–2.41)	0.74	-	-	-	-
***Father***						
*Employment*						
Paid job fulltime	1.00	-	-	-	-	-
Paid job part time	0.70 (0.30–1.65)	0.42	-	-	-	-
No paid job	1.23 (0.43–3.50)	0.70	-	-	-	-
*Educational level*						
Low	1.00	-	-	-	-	-
Intermediate	0.77 (0.44–1.33)	0.35	-	-	-	-
High	0.81 (0.46–1.42)	0.47	-	-	-	-
*Non-Western migrant*	0.54 (0.19–1.50)	0.24	-	-	**0.18 (0.05–0.63)**	**0.01**
**PMT constructs ^a^**						
Self-efficacy (−2, +2)	**0.53 (0.45–0.62)**	**<0.001**	-	-	**0.57 (0.47–0.69)**	**<0.001**
Response efficacy (−2, +2)	**0.26 (0.19–0.35)**	**<0.001**	-	-	**0.34 (0.24–0.48)**	**<0.001**
Vulnerability (−2, +2)	**1.30 (1.05–1.61)**	**0.02**	-	-	-	-
Severity (−2, +2)	**0.63 (0.52–0.76)**	**<0.001**	-	-	**0.74 (0.58–0.93)**	**0.01**

^a ^The OR represents a one-unit change in the scale score.

### 3.3. Discussion

Parents reported leaving their infants unsupervised in the bathtub. These parents expose their infant to the avoidable risk of becoming immersed in water, which could lead to fatal drowning. Since some young children are unable to right themselves if they fall over in the bathtub, they are at great risk of drowning if supervision is absent during bathing. Therefore, effective strategies for drowning prevention, aimed at improved parental supervision during bathing, should be developed.

Our analyses indicate the need for tailoring these strategies to parents with older infants (8–12 months) and parents with low Protection Motivation Theory constructs.

When infants grow older, parents in our study reported they were more likely to leave their infant unsupervised in the bathtub. However, such young children below the age of 12 months may still not be able to prevent themselves from drowning and therefore should never—not even for a moment—be left alone [20].

When parents have two or more children, they are even more likely to leave their infant alone in the bathtub. Reasons for this could be that they are busy with their other child or children, having to leave their infant alone, maybe just for a few moments, not realizing the risk for the infant. It is also possible that parents do not think of the risk of drowning, because nothing has happened before with their infant or older children, so these parents may have considerations like “why would there be a risk now?”. Parents’ may also belief that their child does not need to be supervised all time.

Parents who leave their infant unsupervised in the bathtub reported less self-efficacy, response efficacy and severity. It could be possible that parents do not believe they are able to always supervise their child, do not think supervision could help prevent their child from drowning, and are not aware of the consequences of an injury occurring in the bathtub. Enhancing parents’ knowledge and abilities for never leaving their child unsupervised and explaining why supervision helps to prevent infants from drowning, could increase parental understanding of the importance to supervise their infants in the bathtub. Furthermore, parents need to be better informed about the potential severity of getting injured in the bathtub. Improving these insights could lead to more safe behaviour. Therefore, interventions should aim at enlarging the knowledge about these items.

Various studies have reported inadequate supervision as an important risk factor for drowning in children [3,12,13,21]. A study by Lee *et al*. reported unsupervised bathing by 5% of parents [3]. Our study reports unsupervised bathing of very young children (4–12 months). Furthermore, correlates of unsupervised bathing were determined with this study, which can be used to identify specific target groups of parents in campaigns which promote safe behaviour.

Some limitations of this study need to be addressed. Because our study relied on self-report of supervision during bathing, misclassification could have occurred: for example, parents might have given socially desirable answers. This might result in underestimating the percentage of parents leaving their infant unsupervised in the bathtub.

Participation rate in this study, 46%, was low. There is no data available on the characteristics of parents who did not wish to participate in this study. It is difficult to ascertain whether the associations found would be different in non-responders. Also, it is not clear whether parents who did not wish to participate showed less or more unsafe behaviour with regard to supervision of their infant.

In 93% of participants, it was the mother who responded to the invitation to participate in the survey and completed the questionnaire. In the written information we provided to parents we asked if the parent spending most time with the child could complete the questionnaire. This could explain why most participants were mothers.

We dichotomized the level of supervision, instead of using five different categories, in order to get large enough subgroups for analysis. Although there is a difference in leaving a child unsupervised in the bathtub very often and rarely, the recommendation of the American Academy of Pediatrics is to never leave the child unsupervised [20].

To change the undesirable parental beliefs and, more importantly, parental behaviours, physicians and nurses are crucial in educating parents about the risks and consequences of infants drowning in bathtubs when left unsupervised [20]. Parents need to be informed about the risks and we recommend discussing possible solutions for this issue during the well-child visits.

We used one single question to address the level of supervision during bathing. Further research could be extended with the length of time parents estimated that they left their child alone in the bathtub. This could also be important in order to gain insight in what parents think about the time they can leave a child alone in the bathtub and the reasons for parents to leave their child unsupervised in the bathtub. Furthermore the depth of water the child was left unsupervised in could be addressed. A small child can drown in a few centimetres of water at the bottom of the bathtub [1]. Data can be collected on use of devices in the bathtub, like infant bath seats. Such devices could give a false sense of security and a parent or caregiver might be more likely to leave the child alone in the bathtub [6,13,22]. Therefore, they should not be recommended by health care professionals to use as safety devices. In addition shared bathing can be addressed, where infants are not supervised by adults, but are bathed in the company of an older sibling. This may also be a risk factor with regard to drowning in the bathtub [21].

Furthermore, we recommend theory and evidence-based development of strategies to promote drowning prevention, effect-evaluation of such strategies and wide scale implementation. Future studies could also be extended with home safety observations in order to eliminate possible misclassification. Furthermore, analyses of fatal or nonfatal drowning could give more insight in the validity of parent responses regarding their supervision skills and behaviour.

## 4. Conclusions

Young children are in need of supervision to prevent them from drowning. Strategies for infant drowning prevention in bathtubs should target the following parents: those of older infants, who have more than one child, where the father is a non-Western migrant, who have low self-efficacy, response efficacy and perceived severity in relation to infant bath drowning, since these are important correlates of leaving a child unsupervised in the bathtub.
